# Does Grammatical Number Influence the Semantic Priming Between Number Cues and Words Related to Vertical Space? An Investigation Using Virtual Reality

**DOI:** 10.3389/fpsyg.2018.00573

**Published:** 2018-04-20

**Authors:** Martin Lachmair, Susana Ruiz Fernandez, Peter Gerjets

**Affiliations:** ^1^Leibniz-Institut für Wissensmedien, Tuebingen, Germany; ^2^LEAD Graduate School and Research Network, University of Tuebingen, Tuebingen, Germany; ^3^FOM-Hochschule fuer Oekonomie und Management, Essen, Germany

**Keywords:** numerical cognition, grammatical number, space-number associations, space-word associations, grounded cognition, embodied cognition

## Abstract

The GES framework postulates a hierarchical order between grounded, embodied, and situated representations. Against this background, the present study investigated the relation of two effects: (i) a semantic priming between number cues and words with referents up or down in the world according to the number's magnitude which is supposed to be grounded (cf. Lachmair et al., 2014) and (ii) the compatibility between number cues and the grammatical word form of the words according to the number's multitude which is supposed to be embodied (cf. Roettger and Domahs, 2015). In two experiments words referring to objects up or down in the world and spatially neutral words were presented subsequent to the numbers “1” and “9.” In Experiment 1 words were presented in singular word form and in Experiment 2 in plural word form. For the first time, Virtual Reality was used in such an experimental setup in order to reduce spatial predispositions of participants and to provide a homogeneous experimental environment for replication purposes. According to GES it was expected that the spatial semantic priming should occur in both grammatical word forms. However, the compatibility with grammatical number should only occur for the plural word form due to its markedness. The results of Experiment 1 support the spatial-semantic-priming-hypothesis but not the grammatical-number-hypothesis. The results of Experiment 2 supported only the grammatical-number-hypothesis. It is argued that the grounded spatial effect of Experiment 1 was not affected by grammatical number. However, in Experiment 2 this effect vanished due to an activated embodied reference frame according to grammatical number.

## Introduction

Recent studies suggest a common representational system for numbers and words with referents typically located up or down in the world (Lachmair et al., 2014, 2016). It is suggested that this common representational system integrates meaning attributes of the associated words (e.g., “ground” vs. “sky”) and numbers according to their magnitude (“1,” “2” vs. “8,” “9”) with regard to vertical space. This argumentation relies on the assumption that numbers are ordered in vertical space from the bottom to the top, which has indeed be shown in several studies (e.g., Schwarz and Keus, 2004).

However, space-number associations have also been shown in other spatial dimensions. For example the space-number associated response codes (SNARC) effect assumes that numbers are ordered according to a horizontal mental number line (MNL) associating low numbers to the left and high numbers to the right of an active self (Dehaene et al., 1993; Fischer and Shaki, 2014; for a review see Winter et al., 2015). Interestingly, it has also been shown that grammatical number of words can elicit effects similar to SNARC. This has been found in a study by Roettger and Domahs (2015), indicating an advantage in later stages of responses for singular words (e.g., “lion”) to the left (analog to low numbers) and a disadvantage to the right (analog to high numbers). Importantly, a reversed pattern was observed for words in plural word form (e.g., “lions”).

But considering the cross-domain semantic priming between numbers and words on one hand (cf. Lachmair et al., 2014, 2016) and the interaction of number and word form on the other (cf. Roettger and Domahs, 2015), it would be of interest to investigate if and how magnitude and multitude, which both rely on the cardinality meaning of numbers, would affect the processing of spatially related words in singular or plural word form.

Although the view of embodied cognition can explain cross-domain effects between numbers and spatially related words according to sensorimotor experiences stemming from interaction with the environment, this view makes no concrete predictions about the relation of two interactions, one between spatially related words and magnitude and the other between grammatical number and multitude. Thus, a structure within the view of embodied cognition is needed that allows for more elaborated classifications. Recently, it has been proposed that the experiential source of a spatial association could provide the basis for such a structure (Fischer and Brugger, 2011; Fischer, 2012; Myachykov et al., 2014). This seems plausible, since some of them seem to refer to body characteristics of individuals (e.g., Huber et al., 2015), cultural conventions (e.g., reading direction; e.g., Shaki et al., 2009), or overlearned experiences (e.g., practicing with an instrument; e.g., Stewart et al., 2013) whereas others seem to be context-sensitive (e.g., Bächtold et al., 1998) or even depend on general physical laws (e.g., Lachmair et al., 2014).

Further, this suggests that experiential sources also allow categorizing associations of experiential representations according to their robustness, strength, and flexibility. Support for this view comes from a recent study comparing the behavior on musical pitch between well-trained cello player and non-musicians (Lachmair et al., 2017). On a cello, *high* tones are produced by grasping the strings *downwards* to the tailpiece and *low* tones by grasping the strings *upwards* to the scroll. But typically, high pitch is associated with upper and low pitch with lower vertical space (e.g., Rusconi et al., 2006). Thus, according to embodied cognition, well-trained cello player should show a reversed space-pitch association compared to non-musicians. Indeed, the study found typical space-pitch associations for non-musicians in a piano context (tones presented in piano timbre together with a piano picture) as well as in a cello context (tones presented in cello timbre together with a cello picture). For cello players, the authors also found a typical space-pitch association in the piano context. However, in the cello context this spatial arrangement vanished, but was *not* reversed. It was argued that two reference frames are active when cello player process the pitch of cello tones. One reference frame is rather robust reflecting statistics of natural auditory scenes according to physical laws (cf. Parise et al., 2014) and the other reflects the long-term sensorimotor experiences from practicing with the cello. Their opposing arrangement made the effect disappear.

The so-called GES framework takes such rationales into account; the different experiential sources are denoted with Groundedness, Embodiedness, and Situatedness of mental representations (Fischer, 2012). Accordingly, mental representations are (1) grounded if they reflect the impact of universal physical constraints that shape the evolution of the cognitive system and its physical body over a long period of time. One such physical constraint is earth's gravity which provides an omnipresent stable reference for vertical space. Several findings in the research fields of numerical or language processing can be attributed to that. For example, it has been shown that mental representations of high and low numbers (e.g., Schwarz and Keus, 2004), but also of words associated to referents up (e.g., “roof”) or down (e.g., “root”) in the world can integrate spatial meaning attributes referring to vertical space (e.g., Lachmair et al., 2011). These representations are presumably re-activated during cognitive processing of the active self, facilitating subsequent congruent responses upwards or downwards.

Further, mental representations are (2) embodied if they reflect sensory-motor experience of the individual according to the attributes of the body or cultural conventions. For example several studies suggest that the dominant side of the body is also the side which is associated with positive valence (e.g., Casasanto, 2009). This has been shown for words but also e.g., for valence-laden images moved in horizontal space on a touchscreen (e.g., Cervera Torres et al., 2018). Another example from the research field of numerical cognition is the earlier mentioned SNARC effect (e.g., Dehaene et al., 1993) describing that the numbers from 1 to 9 are horizontally aligned on a continuum, the so-called MNL, from the left side to the right side of an active self. Interestingly, there is also evidence that suggests a reverse alignment of this number line for left-handers (i.e., high numbers are associated with the left and low numbers with the right side; Huber et al., 2015). This is evidence for an embodied spatial alignment according to handedness. These examples show that embodied spatial associations are more flexible and follow the characteristics of the human body or cultural conventions. Compared to Groundedness, they are rather volatile and also easier to retrain.

Beyond Groundedness and Embodiedness, there are (3) also aspects of Situatedness that affect mental representations. For example, the typical horizontal alignment of numbers is canceled when numbers are processed after one had to imagine a clock face (Bächtold et al., 1998). But also the context dependency of the space-pitch association of cello players provides evidence for Situatedness (see above).

Crucially, GES proposes a hierarchical arrangement in which Groundedness is superior to Embodiedness and Situatedness, because of the generality of physical laws suggesting a higher robustness of spatial associations of mental representations. In addition, Embodiedness is superior to Situatedness. This is due to embodied aspects that seem to be more robust than highly volatile situated aspects, which presumably require the highest need of flexibility.

Thus, the purpose of the present study was to test GES by investigating how the cross-domain semantic priming between numbers and spatially related words, which is according to GES grounded, would be affected by the grammatical number of the words which is according to GES embodied.

Therefore, we revisited first the study by Lachmair et al. (2014), focusing on cross-domain semantic priming between numbers and words in singular word form with regard to vertical space. The core of their study was an experiment with a lexical decision task where participants were presented with number-word pairs. A pair consisting of a low number (“1,” “2”) and a down-word (“root”) was considered spatially congruent compared to a low number and an up-word (“roof”) which was considered spatially incongruent. The same held for a high number (“8,” “9”) and an up-word compared to a high number and a down-word. The study found shorter reaction times (RTs) for congruent compared to incongruent number-word pairs.

However, the study made no predictions or drew conclusions toward the word form and the individual number cues. Therefore, it is hard to say if processing of the singular word form is easier subsequent to the number cue “1” compared to the other number cues “2,” “8,” or “9.” Moreover, the singular word form is unmarked (cf. Roettger and Domahs, 2015), making an influence of multitude on the processing of the words rather unlikely. For these reasons, neutral words were introduced in the present study and as number cues only the numbers “1” and “9” were used. With this, it was investigated if the number cue “1” would provide an advantage for neutral nouns in singular word form compared to the number cue “9.”

Concretely, hypotheses were formulated for the spatially related words and the spatially neutral words. For the former, cross-domain semantic priming between low (“1”) and high (“9”) number cues and words referring to objects up or down in the world was expected according to groundedness on vertical space. An influence of grammatical number was not expected because the singular word form is supposed to be unmarked and, according to GES, as an embodied effect inferior to the spatial groundedness (semantic-priming-hypothesis A).

For spatially neutral words two outcomes seem possible: (i) reactions to neutral words are facilitated subsequent to the low number cue “1” compared to the high number cue “9” according to the singular word form, or (ii) reaction times are not affected according to the notion that the singular word form is supposed to be unmarked and grammatical word form is not salient enough due to the lack of the plural word form (grammatical-number-hypothesis A).

## Experiment 1

As described above, participants performed a lexical decision task with *singular* nouns denoting objects that are typically encountered in upper, lower or neutral locations in vertical space (e.g., *roof* vs. *root* vs. *machine*, respectively). These nouns were preceded by either the low number cue “1” or the high number cue “9.” Moreover, in contrast to the previous study by Lachmair et al. (2014), another experimental setting was introduced. Typically, experimental settings in laboratories are full of spatial cues (e.g., tables, edges of pc-monitor, and arrangement of response keys) that may prepare and affect the cognitive system (e.g., Lebois et al., 2015). This may prevent to draw conclusions toward an automatic re-activation of meaning representations and could also lead to an advantage of spatially over non-spatially related hypotheses, in the present study an advantage of the semantic-priming-hypothesis over the grammatical-number-hypothesis. In order to reduce this advantage, we introduced Virtual Reality (VR) as experimental setting.

The main characteristic of VR is that it is presented by a Head Mounted Display (HMD) that separates the visual perception from the surrounding environment. This constitutes the immersive character of VR that produces subjective presence, a feeling of “being there” in the virtual world. At the same time this helps to eliminate the visual spatial cues that typically occur in experimental setups in real world laboratories. Thus, when using the same hardware, VR creates a visually stable environment across laboratories.

### Participants

Twenty seven right-handed native speakers of German (14 female; *M*_*age*_ = 23.86 years, *SD* = 7.18) took part in this experiment. They gave written informed consent and received course credit or financial reimbursement of 8 Euros per hour for participation. All participants had normal or corrected-to-normal vision. The study protocol was approved in advance by the Local Ethics Commission of the Leibniz-Institut für Wissensmedien in Tuebingen.

### Materials and apparatus

Materials consisted of the numbers “1” and “9,” as well as 60 German nouns and 20 pseudo words. Of the 60 nouns, 20 referred to an object that is typically located in upper vertical space, 20 referred to objects that are typically located in lower vertical space, and 20 referred to objects denoting a neutral position according to verticality. All nouns were taken from the study by Lachmair et al. (2011). Thus, they were controlled for frequency, length, and for the typical vertical position of their referent (cf. Lachmair et al., 2011). Words and numbers were presented in white against a black background. Stimuli were displayed with a HTC Vive Head Mounted Display (HMD) with a constant frame rate of 90 Hz. The paradigm was programmed with the gaming engine Unity 5.5.0f3. Responses were recorded with the Vive controllers using the button for the index fingers of the right and left controller as right and left hand responses. The experiment was conducted on a high performing gaming desktop equipped with an Intel Core i7-6700K with 16 GB RAM and an NVidia GeForce GTX 980 Ti graphic card with 6 GB RAM. For those interested in a replication of the study, we provide the employed environment for Unity on Github together with the instructions how to use it.

### Procedure and design

In this experiment participants were presented with singular nouns preceded by a one-digit number cue, either 1 or 9. Cues and subsequent nouns were presented centrally on the HMD. Participant's task was to decide whether the presented letter string was a correct German word or not. Each participant started with a short practice block (32 trials) consisting of words of the word-categories up, down, neutral, and pseudowords presented subsequent to the number cues. Then, in the first half of the experiment, participants had to respond by pressing the left trigger to words and the right trigger to pseudo-words. The second half of the experiment started with another 32 practice trials in which hand-to-response mapping was reversed. Each trial started with a centered fixation cross (500 ms), followed by a number cue presented for 300 ms. Then the (pseudo)-word appeared and stayed on the screen until response.

Response times (RTs) were measured as the time from word onset to a trigger response. Each stimulus was presented eight times (four times in each half), resulting in a total of 640 experimental trials (480 word-trials and 160 pseudo word-trials), subdivided into 8 blocks, separated by self-paced breaks with error information. The design was a 2 × 3 design with the number cues (low, high) and the implicit locational association of words (word category: up, down, neutral) as within- participant factors.

### Results

All data were analyzed using R (R Development Core Team, 2017). Responses to pseudo words were excluded from analyses. A trimming procedure further eliminated responses faster than 200 ms and slower than 3,000 ms (0.75%), erroneous responses (1.36%), as well as responses for which RT deviated by more than 3 SDs from the individual's mean of each combination of conditions. This led to a loss of 2.1% of the data.

**Figure 1:**
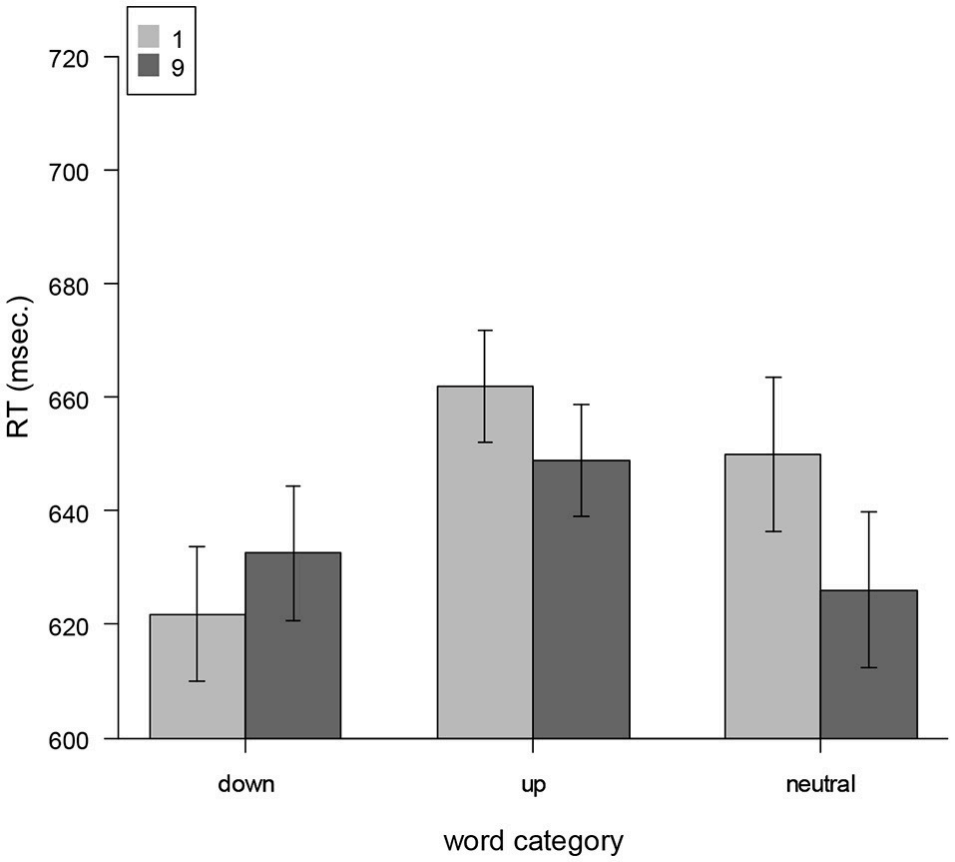
Mean reaction times as a function of implicit locational association of words (up, down, neutral) and numbers (high, low). Error bars represent 95% confidence intervals.

In order to investigate our hypotheses, we conducted an ANOVA with the within-factors *number cue* (low vs. high) and *word category* (up vs. down vs. neutral) as well as their interaction. The ANOVA revealed a significant main effect for word category [*F*_(2, 52)_ = 14.29, *p* < 0.001, ${\eta_{p}}^{2}=$ 0.35] indicating fastest responses to down-words (628 ms), followed by neutral words (639 ms) and up-words (656 ms). Further, for number cue “1” the mean response time was 646 ms compared to 636 ms for the number cue “9.” This difference was not significant [*F*_(1, 26)_ = 2.7, *p* = 0.11]. Interestingly, there was a significant interaction between number cue and the word category [*F*_(2, 52)_ = 4.02, *p* = 0.02, ${\eta_{p}}^{2}=$ 0.13]. This is illustrated in Figure 1. Mean reaction times are described in Table 1. In order to test for the semantic-priming-hypothesis we excluded the neutral words and conducted another ANOVA. This showed again a significant effect of word category [*F*_(1, 26)_ = 24.18, *p* < 0.001] and, more important, a significant interaction between number cue and word category [*F*_(1, 26)_ = 5.33, *p* = 0.03, ${\eta_{p}}^{2}=$ 0.17], indicating shorter reaction times in semantically congruent conditions (i.e., high numbers followed by up-words and low numbers followed by down-words) compared to semantically incongruent conditions (i.e., low numbers followed by up-words and high numbers followed by down-words). Follow-up one-tailed *t*-tests showed a tendency for faster RTs for up-words subsequent to high number cue compared to low number cue [*t*_(26)_ = 1.49, *p* = 0.073]. For down-words the one-tailed *t*-test showed a tendency for faster RTs subsequent to low number cue compared to high number cue [*t*_(26)_ = −1.35, *p* = 0.094]. Taken together, these results support the semantic-priming-hypothesis.

**Table 1:** Mean reaction times.

	**Word category**		
**Number cue**	**Down (ms)**	**Up (ms)**	**Neutral (ms)**
1	623	663	653
9	634	648	626

We also analyzed the simple effect of number cue for neutral words. This effect was significant [*t*_(26)_ = 2.32, *p* = 0.014, ${\eta_{p}}^{2}=$ 0.17]. However, the direction of this effect did not follow the prediction of the grammatical-number-hypothesis.

#### Error analysis

We further conducted an error analysis with trials with incorrect responses. This analysis indicated an almost significant difference of error rates between word categories [down: 1.06%, neutral: 1.06%, up: 1.94%; *F*_(2, 52)_ = 3.14, *p* = 0.052]. The interaction between number cues and word category for error rates, however, was not significant (*F* < 1, *p* = 0.97).

#### Bayesian analyses

Keeping in mind controversies regarding confirmation of the null hypothesis using traditional statistical inference, we additionally conducted a Bayesian analysis by using the R-package “BayesFactor” (Morey and Rouder, 2015). We used the same model than for the ANOVA as full model, i.e., word category (down, neutral, up), number cue (“1,” “9”) and their interaction as fixed factors and participant as random factor with repeated measures. Bayesian factors for all relevant models are displayed in Table 2. Dividing the factor for the interaction-model [4] by the factor of the non-interaction-model [3] (*BF* = 3.86) showed positive evidence for the interaction model according to the criteria of Raftery (1995). We conducted the same analysis only for the semantic-priming-hypothesis with up- and down-words and the number cues. The Bayes Factors are displayed in Table 3. Dividing the Bayesian factor of the interaction-model [4] by the Bayesian factor of the non-interaction model [3] (*BF* = 1.65) showed weak evidence for the interaction model compared to the non-interaction model, according to the criteria of Raftery (1995). The Bayes factor for the grammatical-number-hypothesis for neutral words was *BF* = 1.94, suggested also weak evidence according to Raftery (1995), although not as hypothesized.

**Table 2:** Bayesian factors for the full model.

**Model**	**Bayes factor**
[1] NumberCue + Participant	0.71
[2] WordCategory + Participant	91.73
[3] NumberCue + WordCategory + Participant	81.47
[4] NumberCue + WordCategory + NumberCue:WordCategory + Participant	313.90

**Table 3:** Bayesian factors for the semantic-priming-hypothesis.

**Model**	**Bayes factor**
[1] NumberCue + Participant	0.21
[2] WordCategory + Participant	519.22
[3] NumberCue + WordCategory + Participant	109.06
[4] NumberCue + WordCategory + NumberCue:WordCategory + Participant	180.05

### Discussion

The results of Experiment 1 support the predictions of the semantic-priming-hypothesis, expecting an interaction effect between low and high number cues and up- and down-words according to spatial meaning. This replicates the finding of Lachmair et al. (2014) showing cross-domain semantic priming related to vertical space. Notably, this has been found in an experimental setting using, for the first time, a highly immersive virtual reality technology, where neither the arrangement of response keys nor other spatial cues from the real environment were visible for participants. This supports the view of automatically re-activated mental representations of words and numbers.

With regard to the grammatical-number hypothesis, although the mean reaction time for down-words subsequent to number cue “1” was faster compared to number cue “9,” it is rather unlikely that this advantage can be attributed to the singular word form. Otherwise a similar pattern should have been obtained for the other word categories, at least for spatially neutral words. However, as the analyses showed, this difference was significant in the wrong direction, showing faster reaction times subsequent to number cue “9” compared to number cue “1.”

An explanation of this result could be due to the different experiential sources of magnitude and multitude. Accordingly, it is conceivable that the Groundedness of the spatial compatibility lead to an activation of the magnitude of the number cue also for the neutral words. Thus, these words were not associated with multitude according to their singular word form (which would have been shown faster reaction times subsequent to the number “1” but not to the number “9”), but rather for example according to the size of the word's referents. Accordingly small objects would lead to faster responses subsequent to the number “1” whereas large objects to faster responses subsequent to the number “9.” This hindered grammatical number to become active, which would have been lead to compatibility according to grammatical number. Moreover, the instructions of the experiment did not point to grammatical number in contrast to the study by Roettger and Domahs (2015). Together with the lack of plural word forms in this experiment, this could have been make grammatical number not salient enough in order to affect the responses.

The lack of a consistent difference for all word categories might also lay in the markedness of the singular word form. As stated in the study by Roettger and Domahs (2015), the singular word form is assumed to be the mainly used word form of a language. In contrast to the plural word form it is not overtly coded and presumably less complex. These might be reasons why “singular” as the grammatical word form in this experiment is not promoted and thus, not have been registered by participants. As a consequence, nothing can be said so far toward embodiedness of this cultural convention.

## Experiment 2

In order to get a complete picture for grammatical number of the nouns, in the second experiment the same words were presented; but this time in the plural word form. This should still lead to a spatial semantic priming between high and low number cues and words referring to objects up or down in the world, similar to a study that showed a semantic priming between spatially related words in plural word form and the number cues “2,” “3” as low numbers and “8,” “9” as high numbers (Lachmair et al., 2018; semantic-priming-hypothesis B).

In contrast to the singular word form in Experiment 1, the plural word form is linguistically marked. Markedness is a cultural convention resulting from an asymmetry between two poles based on the polarity correspondence principle (Proctor and Cho, 2006); in this case the singular-plural opposition. As denoted in the study by Roettger and Domahs (2015), the plural word form is marked for several reasons: (i) it is usually derived from the singular word form, (ii) it is typically overtly encoded through an additional word ending (e.g., “roofs,” “roots”), thus rather complex, also in the sense of representing a multitude of something referred to by the word.

As this should be more noticeable for the participants, we expected this time an advantage of word form, specifically for spatially neutral words, subsequent to the number cue “9” compared to the number cue “1” (grammatical-number-hypothesis B).

### Participants

Twenty two right-handed native speakers of German (17 female; *M*_*age*_ = 22.64 years, *SD* = 3.17) took part in this experiment. They gave written informed consent and received course credit or financial reimbursement of 8 Euros per hour for participation. All participants had normal or corrected-to-normal vision. The study protocol was approved in advance by the Local Ethics Commission of the Leibniz-Institut für Wissensmedien in Tuebingen.

### Materials and apparatus

The same material and setting than in Experiment 1 was used. However, the nouns were now presented in plural word form. The pseudo-words were also presented in a “plural” word form by adding an “e” or an “s” at the end of the word.

### Procedure and design

The procedure and design were the same as in Experiment 1.

### Results

All data were analyzed using R (R Development Core Team, 2017). Responses to pseudo words were excluded from analyses, too. A trimming procedure further eliminated responses faster than 200 ms and slower than 3,000 ms (0.99%), erroneous responses (1.57%), as well as responses for which RT deviated by more than 3 *SD*s from the individual's mean of each combination of conditions. This led to a loss of 2.33% of the data. Again, an ANOVA was conducted with the within-factors number cue (low vs. high) and word category (up vs. down vs. neutral) as well as their interaction.

**Figure 2:**
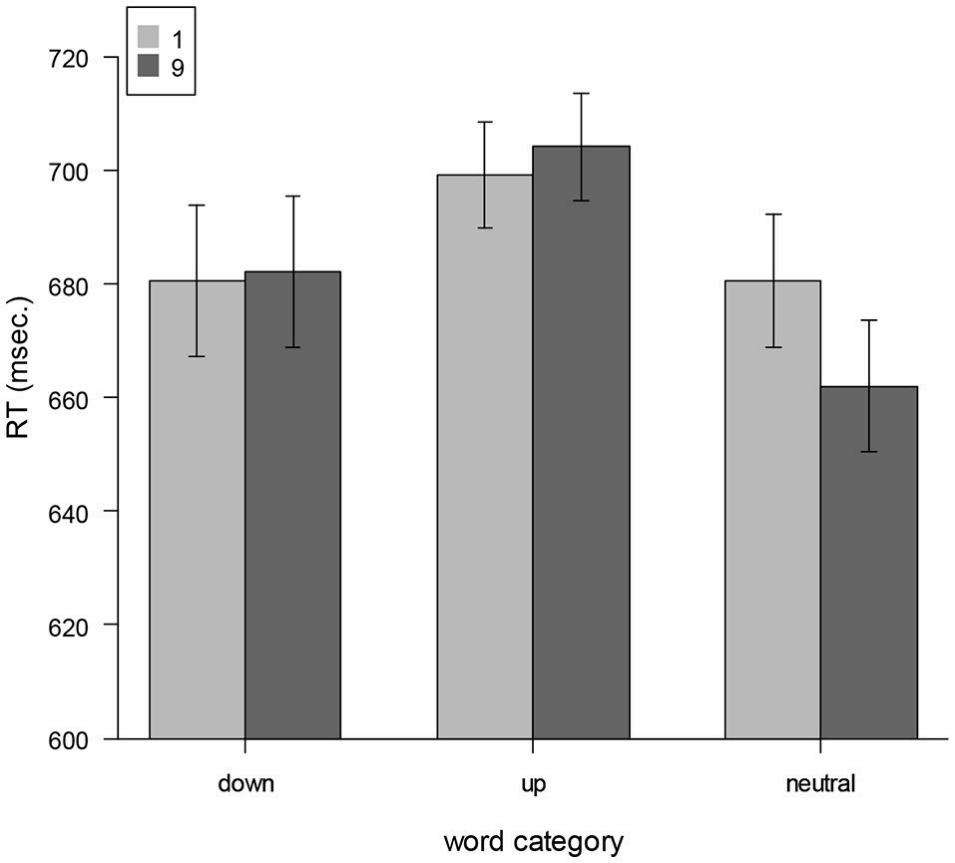
Mean reaction times as a function of implicit locational association of words (up, down, neutral) and numbers (high, low). Error bars represent 95% confidence intervals.

The ANOVA revealed a significant main effect for word category [*F*_(2, 52)_ = 26.82, *p* < 0.001, ${\eta_{p}}^{2}=$ 0.51] indicating fastest responses for neutral words (671 ms), followed by down-words (683 ms) and up-words (702 ms). There was no main effect for number cue (*F* < 1, *p* = 0.39). The interaction between number cue and the word category was also not significant [*F*_(2, 52)_ = 1.72, *p* = 0.19]. This is illustrated in Figure 2. Mean reaction times are described in Table 4.

**Table 4:** Mean reaction times.

	**Word category**		
**Number cue**	**Down (ms)**	**Up (ms)**	**Neutral (ms)**
1	682	700	681
9	684	704	661

To paint the whole picture we conducted the same additional ANOVA than in Experiment 1. The interaction between words with implicit down- and up-ward associations and number cue magnitude by excluding trials with neutral words was not significant [*F*_(1, 26)_ < 1, *p* = 0.88]. Follow-up one-tailed *t*-tests for up- and down-words were also not significant (*ps* > 0.3). Thus, this result did not support the semantic-priming-hypothesis for plural words referring to objects in upper or lower locations. However, the one-tailed *t*-test of number cue for neutral words was significant [*t*_(26)_ = 2.03, *p* = 0.027], indicating faster responses to the high number cue (661 ms) compared to the low number cue (681 ms). This supports the grammatical-number-hypothesis.

#### Error analysis

An error analysis showed no significant difference of error rates between word categories [*F*_(2, 52)_ = 2.32, *p* = 0.11] and number cues [*F*_(1, 26)_ < 1, *p* = 0.7]. The interaction between number cues and word category for error rates was also not significant (*F* < 1, *p* = 0.43).

#### Bayesian analyses

Again, we conducted a Bayesian analysis analog to Experiment 1. We used the same model than for the ANOVA as full model, i.e., word category (down, neutral, up), number cue (“1,” “9”) and their interaction as fixed factors and participant as random factor with repeated measures. Bayesian factors for all relevant models are displayed in Table 5. Dividing the factor for the interaction-model [4] by the factor of the non-interaction-model [3] (*BF* = 0.79) suggested no evidence for the interaction model according to the criteria of Raftery (1995).

**Table 5:** Bayesian factors for the full model.

**Model**	**Bayes factor**
[1] NumberCue + Participant	0.23
[2] WordCategory + Participant	4,442.27
[3] NumberCue + WordCategory + Participant	1,049.10
[4] NumberCue + WordCategory + NumberCue:WordCategory + Participant	834.28

We conducted the same analysis only for the spatial-compatibility-hypothesis with up- and down-words and the number cues. The Bayes Factors are displayed in Table 6. Dividing the factor for the interaction-model [4] by the factors of the other models (*BF* = 0.29) suggest no evidence for the interaction model compared to the non-interaction model [3], according to the criteria of Raftery (1995). The Bayes factor for the grammatical-number-hypothesis for neutral words was *BF* = 1.18, suggesting weak evidence according to Raftery (1995).

**Table 6:** Bayesian factors for the semantic-priming-hypothesis.

**Model**	**Bayes factor**
[1] NumberCue + Participant	0.22
[2] WordCategory + Participant	12.81
[3] NumberCue + WordCategory + Participant	2.89
[4] NumberCue + WordCategory + NumberCue:WordCategory + Participant	0.84

### Discussion

Testing the semantic-priming-hypothesis for Experiment 2 showed no interaction between number cues and up- and down-words. Bayesian analysis confirmed no evidence for this interaction and therefore no support for the spatial-compatibility effect as shown for example in the study by Lachmair et al. (2018). One reason for that might be that in contrast to the present study the latter study used only “plural” number cues, namely “2,” “3,” “8,” and “9.” This might lead to a general congruency between number cues and word form, which make the spatial semantic congruency between the number cues and the words more salient.

Testing the grammatical-number-hypothesis for Experiment 2 showed a significant difference between the number cues “1” and “9” and the plural word form of the neutral nouns, showing faster reaction times for the high number cue compared to the low number cue. Thus, in contrast to Experiment 1 the difference showed this time in the right direction with faster reaction times of plural word form subsequent to the number 9. In addition, Bayesian analysis showed weak evidence for this hypothesis.

Again, the grammatical word form was not mentioned in the instructions of Experiment 2. However, due to its markedness, the plural word form become more salient compared to Experiment 1 (cf. Berent et al., 2005). This is supported by the neutral words suggesting that the plural word form was recognized by participants leading to an activation of embodied representations of multitude. This could have led to conflicting reference frames, one grounded reference frame according to spatial attributes and one embodied reference frame according to grammatical number (cf. Wood et al., 2006; Lachmair et al., 2017). Accordingly, the spatial semantic priming vanished.

## General discussion

In two experiments GES was tested *by* investigating the cross-domain semantic priming between low and high number cues and words with referents up or down in the world according to the number's magnitude (cf. Lachmair et al., 2014) which is according GES grounded and the compatibility between low and high number cues and the grammatical word form (singular or plural) of the words according to the number's multitude (cf. Roettger and Domahs, 2015) which is according to GES embodied. In order to eliminate typical spatial cues from the experimental environment in laboratories (e.g., arrangement of response keys), for the first time this paradigm was conducted in a highly immersive virtual reality.

In Experiment 1, the semantic priming has been shown for singular nouns referring to objects up or down in the world. However grammatical number had no effect on reaction times, specifically for spatially neutral words. This can be explained with an active grounded reference frame for spatial semantic priming which is not interfered by grammatical number. However using plural word form in Experiment 2, neutral words showed an effect of grammatical number. At the same time the semantic priming vanished. It is argued that, in contrast to Experiment 1, a second embodied reference frame according to grammatical number became active interfering with the grounded reference frame causing the semantic priming to vanish. Further studies should make the grammatical word form more salient for participants in order to test its limits.

## Author contributions

ML: conception, design, programming, statistical analysis, and first draft. SRF, PG, and ML: manuscript revision, read and approved the submitted version.

### Conflict of interest statement

The authors declare that the research was conducted in the absence of any commercial or financial relationships that could be construed as a potential conflict of interest.

## References

[B1] Bächtold D. Baumüller M. Brugger P. (1998). Stimulus-response compatibility in representational space. Neuropsychologia 36, 731–735. 10.1016/S0028-3932(98)00002-5 9751438

[B2] Berent I. Pinker S. Tzelgov J. Bibi U. Goldfarb L. (2005). Computation of semantic number from morphological information. J. Mem. Lang. 53, 342–358. 10.1016/j.jml.2005.05.002

[B3] Casasanto D. (2009). Embodiment of abstract concepts: good and bad in right-and left-handers. J. Exp. Psychol. 138, 351–367. 10.1037/a0015854 19653795

[B4] Cervera Torres S. Fernández S. R. Lachmair M. Gerjets P. (2018). Coding valence in touchscreen interactions: hand dominance and lateral movement influence valence appraisals of emotional pictures. Psychol. Res. [Epub ahead of print]. 10.1007/s00426-018-0971-1 29330594

[B5] Dehaene S. Bossini S. Giraux P. (1993). The mental representation of parity and number magnitude. J. Exp. Psychol. 122, 371–396. 10.1037/0096-3445.122.3.371

[B6] Fischer M. H. (2012). A hierarchical view of grounded, embodied, and situated numerical cognition. Cogn. Process. 13, 161–164. 10.1007/s10339-012-0477-5 22802036

[B7] Fischer M. H. Brugger P. (2011). When digits help digits: spatial–numerical associations point to finger counting as prime example of embodied cognition. Front. Psychol. 2:260. 10.3389/fpsyg.2011.00260 22028696 PMC3198540

[B8] Fischer M. H. Shaki S. (2014). Spatial associations in numerical cognition—From single digits to arithmetic. Q. J. Exp. Psychol. 67, 1461–1483. 10.1080/17470218.2014.927515 25051273

[B9] Huber S. Klein E. Graf M. Nuerk H. C. Moeller K. Willmes K. (2015). Embodied markedness of parity? Examining handedness effects on parity judgments. Psychol. Res. 79, 963–977. 10.1007/s00426-014-0626-9 25394996

[B10] Lachmair M. Cress U. Fissler T. Kurek S. Leininger J. Nuerk H. C. (2017). Music-space associations are grounded, embodied and situated: examination of cello experts and non-musicians in a standard tone discrimination task. Psychol. Res. [Epub ahead of print]. 10.1007/s00426-017-0898-y 28744607 PMC6557872

[B11] Lachmair M. Dudschig C. De Filippis M. de la Vega I. Kaup B. (2011). Root versus roof: automatic activation of location information during word processing. Psychon. Bull. Rev. 18, 1180–1188. 10.3758/s13423-011-0158-x 21913000

[B12] Lachmair M. Dudschig C. de la Vega I. Kaup B. (2014). Relating numeric cognition and language processing: do numbers and words share a common representational platform? Acta Psychol. 148, 107–114. 10.1016/j.actpsy.2013.12.004 24509403

[B13] Lachmair M. Ruiz Fernández S. Gerjets P. (2016). Priming effects between spatial meaning of verbs and numbers are modulated by time intervals: early interference and late facilitation. Can. J. Exp. Psychol. 70, 295–300. 10.1037/cep0000085 27228334

[B14] Lachmair M. Ruiz Fernández S. Moeller K. Nuerk H.-C. Kaup B. (2018). Magnitude or multitude—what counts? Front. Psychol. 9:522. 10.3389/fpsyg.2018.00522 29706917 PMC5906736

[B15] Lebois L. A. Wilson-Mendenhall C. D. Barsalou L. W. (2015). Are automatic conceptual cores the gold standard of semantic processing? The context-dependence of spatial meaning in grounded congruency effects. Cogn. Sci. 39, 1764–1801. 10.1111/cogs.12174 25243925

[B16] Morey R. D. Rouder J. N. (2015). BayesFactor: Computation of Bayes Factors for Common Designs. R package version 0.9.12-2. Available online at: https://CRAN.R-project.org/package=BayesFactor

[B17] Myachykov A. Scheepers C. Fischer M. H. Kessler K. (2014). TEST: a tropic, embodied, and situated theory of cognition. Top. Cogn. Sci. 6, 442–460. 10.1111/tops.12024 23616259

[B18] Parise C. V. Knorre K. Ernst M. O. (2014). Natural auditory scene statistics shapes human spatial hearing. Proc. Natl. Acad. Sci. 111, 6104–6108. 10.1073/pnas.1322705111 24711409 PMC4000839

[B19] Proctor R. W. Cho Y. S. (2006). Polarity correspondence: a general principle for performance of speeded binary classification tasks. Psychol. Bull. 132:416. 10.1037/0033-2909.132.3.416 16719568

[B20] Raftery A. E. (1995). Bayesian model selection in social research. Sociol. Methodol. 25, 111–163. 10.2307/271063

[B21] R Development Core Team (2017). R: A Language and Environment for Statistical Computing. Vienna: R Foundation for Statistical Computing Available online at: https://www.R-project.org/

[B22] Roettger T. B. Domahs F. (2015). Grammatical number elicits SNARC and MARC effects as a function of task demands. Q. J. Exp. Psychol. 68, 1231–1248. 10.1080/17470218.2014.979843 25384199

[B23] Rusconi E. Kwan B. Giordano B. L. Umilta C. Butterworth B. (2006). Spatial representation of pitch height: the SMARC effect. Cognition 99, 113–129. 10.1016/j.cognition.2005.01.004 15925355

[B24] Schwarz W. Keus I. M. (2004). Moving the eyes along the mental number line: comparing SNARC effects with saccadic and manual responses. Attention Percept. Psychophys. 66, 651–664. 10.3758/BF03194909 15311664

[B25] Shaki S. Fischer M. H. Petrusic W. M. (2009). Reading habits for both words and numbers contribute to the SNARC effect. Psychon. Bull. Rev. 16, 328–331. 10.3758/PBR.16.2.328 19293102

[B26] Stewart L. Verdonschot R. G. Kajihara T. Sparks J. (2013). Action-perception coupling in violinists. Front. Hum. Neurosci. 7:349. 10.3389/fnhum.2013.00349 23908612 PMC3726832

[B27] Winter B. Matlock T. Shaki S. Fischer M. H. (2015). Mental number space in three dimensions. Neurosci. Biobehav. Rev. 57, 209–219. 10.1016/j.neubiorev.2015.09.005 26365108

[B28] Wood G. Nuerk H. C. Willmes K. (2006). Crossed hands and the Snarc effect: a failure to replicate Dehaene, Bossini and Giraux (1993). Cortex 42, 1069–1079. 10.1016/S0010-9452(08)70219-3 17209413

